# Delayed Diagnosis of Native-Valve Infective Endocarditis Presenting as Progressive Aortic Regurgitation in Recurrent Methicillin-Sensitive Staphylococcus aureus Bacteraemia With Initially Negative TTE and FDG PET-CT

**DOI:** 10.7759/cureus.108922

**Published:** 2026-05-15

**Authors:** Aldo Zambrano Bardellini

**Affiliations:** 1 Acute Medicine, Royal Free Hospital, London, GBR

**Keywords:** infective endocarditis, mssa bacteraemia, mssa endocarditis, persistent mssa bacteraemia, pet-ct scan

## Abstract

Infective endocarditis (IE) remains a life-threatening condition in which early diagnosis is essential to prevent valvular destruction and systemic complications. Persistent *Staphylococcus aureus* bacteraemia (SAB) is strongly associated with endocardial infection, yet diagnosis may be challenging when initial imaging is inconclusive.

We describe a case of a 55-year-old woman who presented with fever, vomiting, and transient loss of consciousness. Blood cultures grew methicillin-sensitive *Staphylococcus aureus *(MSSA). Initial transthoracic echocardiography (TTE) and positron emission tomography-computed tomography (PET-CT) imaging failed to identify endocarditis. Despite appropriate antimicrobial therapy, she developed recurrent bacteraemia, ongoing pyrexia, and clinical deterioration. Transoesophageal echocardiography (TOE) subsequently demonstrated severe aortic regurgitation consistent with IE. Because of significant underlying liver disease, surgery was deemed prohibitive, and she was managed with prolonged intravenous antibiotics and cardiology follow-up for potential future valve intervention.

This case highlights the importance of maintaining a high clinical suspicion for IE in patients with persistent SAB. It supports guideline recommendations for early TOE when clinical suspicion remains high despite a negative transthoracic study. The final Duke-International Society for Cardiovascular Infectious Diseases (ISCVID) 2023 classification of the case aligned with definite IE. The patient completed six weeks of antibiotic treatment after blood culture clearance, which was achieved at week two following the start of antibiotic therapy.

## Introduction

Infective endocarditis (IE) is a life-threatening condition associated with significant morbidity and mortality, with reported in-hospital mortality rates up to 19.3%, with six-month mortality estimated around 22-24% despite advances in antimicrobial therapy and surgical management [1,2]. Early diagnosis remains challenging due to heterogeneous clinical presentations and the limited sensitivity of initial diagnostic investigations.

*Staphylococcus aureus *bacteraemia (SAB) is a strong risk factor for IE and is associated with a high incidence of endocardial involvement [3,4]. Consequently, both the European Society of Cardiology (ESC) and British Society for Antimicrobial Chemotherapy recommend systematic evaluation for IE in all patients with SAB, including appropriate imaging and clinical assessment [1,5].

Transthoracic echocardiography (TTE) is recommended as the initial imaging modality due to its availability and non-invasive nature; however, its sensitivity for detecting vegetations is limited, particularly in early disease or in patients with suboptimal imaging windows, with reported sensitivities of approximately 70% [1]. In contrast, transoesophageal echocardiography (TOE) provides superior image resolution and diagnostic accuracy, with sensitivities approaching 96%, and is recommended when clinical suspicion remains high despite a negative TTE [6].

Adjunctive imaging modalities such as fluorodeoxyglucose (FDG) positron emission tomography-computed tomography (PET-CT) have emerged as useful tools in selected cases, particularly in prosthetic valve endocarditis, with a sensitivity of 73-86% [7]. However, their diagnostic utility is significantly lower for native valve disease, with a pooled sensitivity of 31% [8,9].

This case illustrates the diagnostic complexity of IE in the context of persistent SAB and initially negative imaging, highlighting the importance of repeat imaging and sustained clinical vigilance in high-risk patients. 

## Case presentation

A 55-year-old woman presented to the emergency department with fever, vomiting, and a fall associated with transient loss of consciousness. Her past medical history was significant for alcohol-related liver disease with portal hypertension and grade I oesophageal varices identified on previous oesophago-gastro-duodenoscopy (OGD). She was classified as Child-Pugh class B, with a United Kingdom Model for End-Stage Liver Disease (UKELD) score of 50 and a Model for End-Stage Liver Disease (MELD) score of 9 on admission [10-12].

On presentation, she was haemodynamically stable but febrile, with markedly raised inflammatory markers, including a C-reactive protein (CRP) of 125 mg/L (reference range <5 mg/L). Initial blood investigations demonstrated a mildly elevated alanine aminotransferase (ALT) of 63 IU/L (reference range <50 IU/L), international normalised ratio (INR) of 1.3, albumin of 30 g/L, and ammonia level of 46 µmol/L, while bilirubin remained within normal limits (Table 1).

**Table 1:** Relevant blood tests on admission and discharge Hb: haemoglobin; WBC: white blood cells; PLT: platelets; INR: international normalised ratio; eGFR: estimated glomerular filtration rate; K: potassium; Na: sodium; ALT: alanine aminotransferase; AST: aspartate aminotransferase; CRP: C-reactive protein

Parameter	On admission	On discharge
Hb (g/L)	125	120
WBC (10⁹/L)	8	7
Neutrophils (10⁹/L)	5	5
PLT (10⁹/L)	181	195
INR	1.3	1.2
Creatinine (µmol/L)	114	68
eGFR (mL/min)	46	90
K (mmol/L)	3.2	4.2
Na (mmol/L)	137	142
Albumin (g/L)	30	35
Bilirubin (µmol/L)	19	22
ALT (IU/L)	63	51
AST (IU/L)	47	45
CRP (mg/L)	125	12
Ammonia (µmol/L)	46	Not performed

Given the history of transient loss of consciousness and fall, a computed tomography (CT) of the head was performed, which demonstrated no acute intracranial abnormalities. There were no focal neurological deficits on examination.

Further investigations revealed methicillin-sensitive *Staphylococcus aureus *(MSSA) bacteraemia isolated in blood cultures. Consequently, the patient was commenced on targeted intravenous cefazolin 2 g every eight hours, in accordance with microbiology recommendations [1].

Indwelling intravascular lines/catheters, intravenous drug use, recent dental procedures, and skin, soft tissue, and urinary tract sources were specifically investigated and excluded based on the clinical history, physical examination, microbiological investigations, and relevant imaging/laboratory studies. A diagnostic paracentesis was considered to rule out spontaneous bacterial peritonitis. However, there was no significant fluid in the abdomen to safely perform a tap. No identifiable source of bacteraemia/infection was identified following this evaluation.

In addition, peripheral stigmata of IE were specifically assessed. There was no evidence of Osler nodes, Janeway lesions, splinter haemorrhages, Roth spots, or conjunctival petechiae on examination.

A subsequent CT of the abdomen and pelvis demonstrated a known cirrhotic liver, mild bilateral pleural effusions with subpleural atelectatic changes, as well as a small volume of free fluid within the pelvis. 

**Figure 1:**
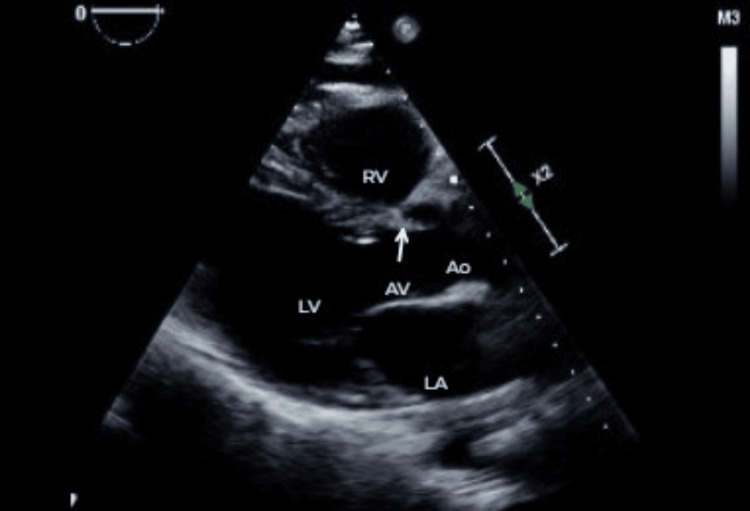
Transthoracic echocardiogram: Parasternal long-axis (PLAX) view demonstrating no obvious vegetations. RV: right ventricle; Ao: ascending aorta; AV: aortic valve; LV: left ventricle; LA: left atrium

**Figure 2:**
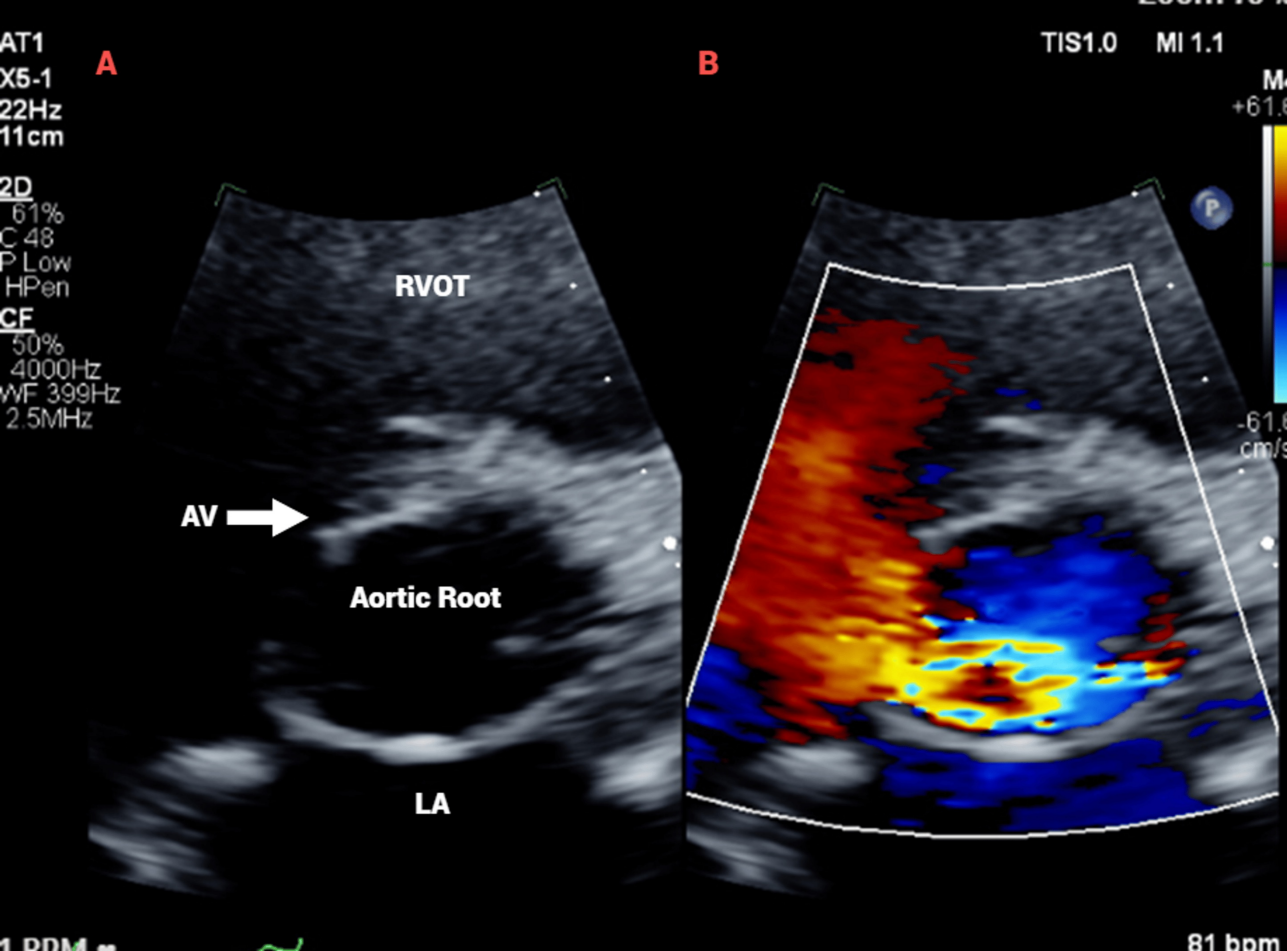
Transthoracic echocardiogram: Parasternal short-axis (PSAX) view at the level of the aortic valve (AV). (A) showing structures at the level of the AV. (B) showing structures with added colour Doppler consistent with moderate aortic regurgitation; no vegetations were noted. Left ventricular internal dimension at end-diastole (LVIDd) was 4.6 cm, and left ventricular ejection fraction (LVEF) was estimated at 55-60%. Vena contracta width measured 5 mm, with no holodiastolic flow reversal in the descending aorta. LA: left atrium; RVOT: right ventricular outflow tract

As a result of a positive MSSA blood culture with no clear source of infection, a TTE was performed. Initial TTE did not demonstrate vegetations but moderate aortic regurgitation (Figures 1-2). A subsequent blood culture on day three was negative.

On day eight following initiation of antibiotic therapy, the patient developed haemodynamic instability, characterised by hypotension, tachycardia, and persistent pyrexia. Supportive management was commenced alongside close clinical monitoring. In discussion with the microbiology team, linezolid 600 mg every 12 hours was added to the existing antibiotic regimen. A repeat set of blood cultures was obtained, which demonstrated recurrent MSSA bacteraemia.

**Figure 3:**
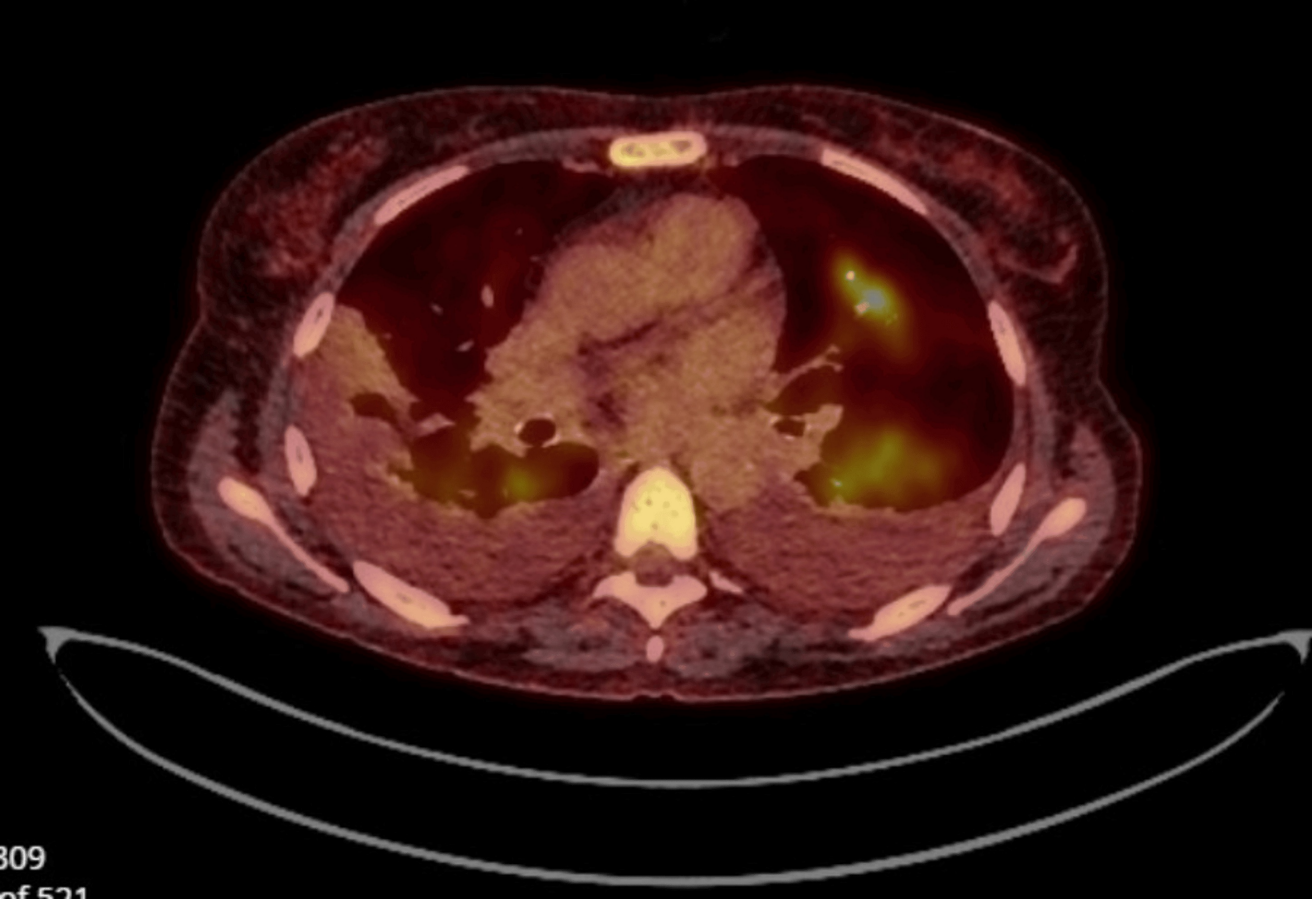
Positron emission tomography (PET) scan demonstrating no abnormal cardiac uptake to suggest endocarditis. Bilateral pleural effusions were present.

In the presence of ongoing sepsis without an identifiable source, PET-CT imaging was performed, reporting satisfactory suppression of normal myocardial uptake and no evidence of abnormal cardiac uptake to suggest endocarditis, but bilateral pleural effusions (Figure 3). Preparation for the scan included fasting for at least six hours prior to the scan. Blood glucose was 4.6 mmol/L at the start of the investigation with an uptake time of 70 min.

The pleural effusions were thought to be predominantly transudative in nature, likely due to a combination of hypoalbuminaemia during admission and continued intravenous fluid requirements in the context of haemodynamic instability. No pleural tap was performed. 

**Figure 4:**
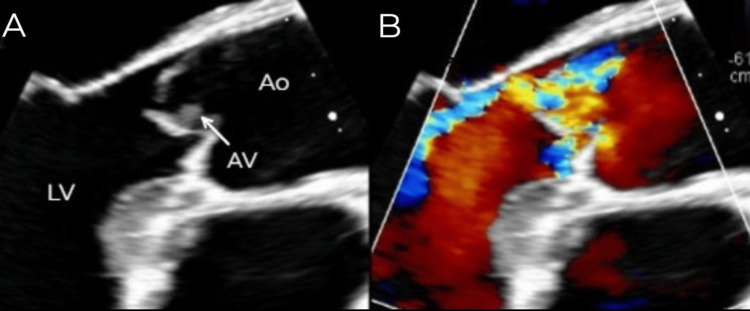
Transoesophageal echocardiogram. (A) Structural view demonstrating the aorta (Ao), aortic valve (AV), and left ventricle (LV). (B) Colour Doppler view demonstrating a broad regurgitant jet consistent with severe aortic regurgitation.

**Figure 5:**
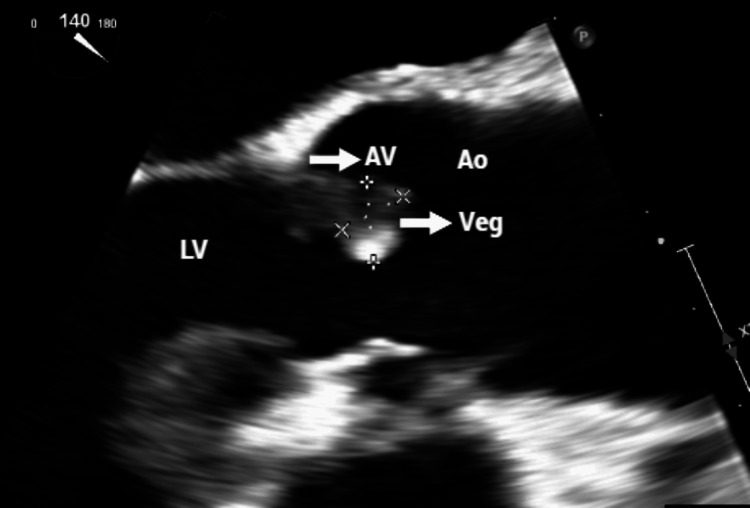
Transoesophageal echocardiogram demonstrating a mobile structure on the left coronary cusp of the aortic valve (AV) measuring 0.5 × 0.4 cm, consistent with vegetation (Veg). No abscess was identified. Ao: ascending aorta; LV: left ventricle

Given the persistent high clinical suspicion for IE, a TOE was performed. This demonstrated a regurgitant jet towards the anterior mitral valve in colour Doppler, consistent with severe aortic regurgitation, and a mobile structure on the left coronary cusp measuring 0.5 x 0.4 cm, indicative of IE of the native valve (Figures 4-5). The interval duration between the TTE and TOE was 10 days. 

The patient was discussed at the endocarditis multidisciplinary team (MDT) meeting, involving specialists from cardiology, cardiothoracic surgery, infectious diseases, and microbiology, with additional input from hepatology. Owing to significant underlying liver disease with portal hypertension, she was considered high risk for valve surgery (Child-Pugh class B, UKELD score 50, MELD score 9). Therefore, she was also deemed unsuitable for liver transplantation due to ongoing alcohol consumption. A decision was made to proceed with prolonged medical therapy.

Linezolid was discontinued after two weeks due to thrombocytopenia, and treatment was continued with cefazolin 2 g three times daily. She completed a six-week course of intravenous antibiotics following blood culture clearance, with three sets of blood cultures remaining negative on day 14. This was followed by clinical and biochemical improvement, including improvement of inflammatory marker levels and haemodynamic stabilisation. Low-dose loop diuretic therapy was subsequently initiated. 

Reassessment for potential transcatheter aortic valve intervention will be undertaken in the outpatient cardiology clinic following repeated echocardiography.

## Discussion

This case highlights several important considerations in the diagnosis and management of IE. Firstly, it reinforces the well-established association between SAB and IE, particularly in the context of recurrent MSSA bacteraemia, where a high index of suspicion should be maintained despite initially non-diagnostic investigations.

Secondly, this case demonstrates the limitations of initial imaging. While TTE is widely available and non-invasive, its sensitivity for detecting vegetations is limited, particularly in early disease, small vegetations, or in patients with poor acoustic windows. In contrast, TOE provides superior resolution and sensitivity and is recommended when clinical suspicion remains high despite a negative TTE [1]. The delayed diagnosis in this patient, despite an initial negative TTE, highlights the importance of repeat imaging in high-risk clinical scenarios such as recurrent MSSA bacteraemia. Furthermore, this case illustrates the dynamic and rapidly progressive nature of IE, with interval imaging demonstrating progression from moderate aortic regurgitation on initial TTE to severe aortic regurgitation on TOE performed 10 days later, accompanied by haemodynamic deterioration.

According to the 2023 Duke-International Society for Cardiovascular Infectious Diseases (ISCVID) criteria, this patient fulfilled the definition of definite IE, with one major microbiological criterion (typical organism - MSSA - isolated from ≥2 blood cultures obtained separately in time), one major imaging criterion (TOE demonstrating valvular vegetation), and two minor criteria (fever and aortic regurgitation) [13].

Thirdly, this case highlights the limitations of FDG PET-CT in native valve endocarditis. While PET-CT has demonstrated high diagnostic accuracy in prosthetic valve and device-related infections, its sensitivity is significantly reduced in native valve IE, with a pooled sensitivity of approximately 31%, leading to potential false-negative findings [1,9]. This underscores the importance of interpreting imaging findings within the broader clinical context and avoiding over-reliance on negative advanced imaging when clinical suspicion remains high.

In addition, this case emphasises the complexity of management decisions in patients with IE and significant comorbid liver disease. Severe aortic regurgitation is generally considered an indication for early surgical intervention; however, advanced chronic liver disease and portal hypertension substantially increase perioperative mortality risk. Reported short-term mortality following cardiac surgery increases significantly across Child-Pugh classes, reaching 9.0% (95% CI 6.6-12.2%) in Child-Pugh A, 37.7% (95% CI 30.8-44.3%) in Child-Pugh B, and 52.0% (95% CI 33.5-70.0%) in Child-Pugh C patients [14]. In this patient, ongoing alcohol consumption also precluded consideration for liver transplantation, limiting therapeutic options.

Finally, this case underscores the importance of multidisciplinary management in complex IE cases. Current ESC guidelines emphasise the value of a dedicated multidisciplinary “endocarditis team” involving cardiology, cardiothoracic surgery, infectious diseases, microbiology, and other relevant specialities to individualise management decisions [1]. In this case, additional hepatology input was essential in balancing the risks and benefits of surgical versus conservative management, ultimately leading to a decision to pursue prolonged medical therapy.

## Conclusions

This case highlights the diagnostic and management challenges of native-valve IE in the setting of recurrent MSSA bacteraemia and advanced liver disease. It emphasises the importance of maintaining a high index of suspicion for IE despite initially negative imaging findings, particularly in high-risk patients. In cases of persistent clinical suspicion and negative TTE, TOE should be performed promptly without unnecessary delay. Furthermore, although FDG PET-CT may provide additional diagnostic information, its limited sensitivity in native-valve IE means that a negative study does not exclude the diagnosis. This case also reinforces the value of applying the 2023 Duke-ISCVID criteria systematically in all suspected cases of IE. Finally, in patients with significant cirrhosis, surgical risk should be carefully quantified using validated scoring systems such as the Child-Pugh and MELD/UKELD scores, with early involvement of hepatology as part of the multidisciplinary endocarditis team to guide individualised management decisions.
